# Drug-Inducible Gene Therapy Effectively Reduces Spontaneous Seizures in Kindled Rats but Creates Off-Target Side Effects in Inhibitory Neurons

**DOI:** 10.3390/ijms241411347

**Published:** 2023-07-12

**Authors:** Kyle A. Sullivan, Iuliia Vitko, Kathryn Blair, Ronald P. Gaykema, Madison J. Failor, Jennifer M. San Pietro, Deblina Dey, John M. Williamson, Ruth L. Stornetta, Jaideep Kapur, Edward Perez-Reyes

**Affiliations:** 1 Department of Pharmacology, University of Virginia, Charlottesville, VA 22980, USA; 2 Computational and Predictive Biology, Oak Ridge National Laboratory, Oak Ridge, TN 37830, USA; 3 Department of Neurology, University of Virginia, Charlottesville, VA 22980, USAjk8t@virginia.edu (J.K.); 4 UVA Brain Institute, University of Virginia, Charlottesville, VA 22980, USA

**Keywords:** gene therapy, adeno-associated virus (AAV), drug-inducible, temporal lobe epilepsy, electroencephalogram (EEG), spontaneous limbic seizures

## Abstract

Over a third of patients with temporal lobe epilepsy (TLE) are not effectively treated with current anti-seizure drugs, spurring the development of gene therapies. The injection of adeno-associated viral vectors (AAV) into the brain has been shown to be a safe and viable approach. However, to date, AAV expression of therapeutic genes has not been regulated. Moreover, a common property of antiepileptic drugs is a narrow therapeutic window between seizure control and side effects. Therefore, a long-term goal is to develop drug-inducible gene therapies that can be regulated by clinically relevant drugs. In this study, a first-generation doxycycline-regulated gene therapy that delivered an engineered version of the leak potassium channel *Kcnk2* (TREK-M) was injected into the hippocampus of male rats. Rats were electrically stimulated until kindled. EEG was monitored 24/7. Electrical kindling revealed an important side effect, as even low expression of TREK M in the absence of doxycycline was sufficient to cause rats to develop spontaneous recurring seizures. Treating the epileptic rats with doxycycline successfully reduced spontaneous seizures. Localization studies of infected neurons suggest seizures were caused by expression in GABAergic inhibitory neurons. In contrast, doxycycline increased the expression of TREK-M in excitatory neurons, thereby reducing seizures through net inhibition of firing. These studies demonstrate that drug-inducible gene therapies are effective in reducing spontaneous seizures and highlight the importance of testing for side effects with pro-epileptic stressors such as electrical kindling. These studies also show the importance of evaluating the location and spread of AAV-based gene therapies in preclinical studies.

## 1. Introduction

Epilepsy is a major neurological disorder with significant economic and human burdens. The National Institute of Neurological Disorders and Stroke (NINDS) estimates there are 3 million Americans with epilepsy, and approximately 0.6 million Americans have temporal lobe epilepsy (TLE; [1]). Despite the size of this problem, medical treatment of TLE fails for roughly a third of patients due to either ineffectiveness, development of pharmacoresistance, or intolerance to side effects [2,3]. Since the 1950s, the only option left for some of these patients has been surgical resection of the temporal lobe and there are several drawbacks to this procedure. First, only about a third of these patients are eligible for surgery [4]. Second, most patients continue to be treated with antiepileptic drugs after surgery [5]. Third, surgery is only effective in controlling seizures in two-thirds of patients, and after 5 years, only half remain seizure-free [6,7]. Fourth, surgery disrupts memory in over one-third of patients [8,9]. Finally, a survey revealed over half of eligible patients believe surgery is too dangerous and want to avoid permanent side effects such as memory impairment [10,11]. Clearly, there is a great need to develop less traumatic therapies. Gene therapy could, therefore, provide a major improvement in the medical treatment of TLE and might be extended to recurrent status epilepticus, focal cortical dysplasia, and focal epilepsies caused by traumatic brain injury.

AAV-based gene therapies have been shown to be clinically effective [12], but none are approved for epilepsy. Many studies have explored the use of gene therapies for epilepsy using proof-of-concept study designs (reviewed in [13]). One promising approach that reached the preclinical study design phase used AAV delivery of the potassium channel *KCNA1* [14,15], or delivery of a CRISPR that stimulated *KCNA1* expression from its endogenous promoter [16]. Although these studies showed about a 50% decrease in seizure frequency, the success of these studies was hampered by inadequate animal models. Key problems with these models include a lack of predictive validity [17], low spontaneous seizure frequencies with sporadic clustering [18,19] and progression of seizure frequencies [14,20].

Current antiepileptic drug therapies reduce seizure activity by either increasing the activity of inhibitory neurons or decreasing the activity of excitatory neurons. In addition to excitatory neurons, cell death of parvalbumin (Parv)-expressing inhibitory neurons has been previously implicated in TLE rodent models [21]. Finding the right drug and dose regimen is a considerable challenge for effectively managing seizures without causing side effects such as sedation and ataxia. Therefore, we focused on developing a drug-inducible gene therapy using tetracycline/doxycycline (Dox)-regulated expression pioneered by the late Hermann Bujard [22]. Two systems were developed, Dox-Off (expression in the absence of doxycycline) and Dox-On (expression with doxycycline present). We reasoned that patient compliance would be higher if they took doxycycline to feel better (Dox-On) rather than to avoid side effects (Dox-Off), so we used molecular cloning techniques to construct a first-in-class Dox-On therapeutic gene therapy. Furthermore, doxycycline is a common antibiotic in human patients, and therefore, increases the translatability of this AAV gene therapy. In this study, we describe the engineering of such an AAV-based, Dox-On system that delivers a modified *Kcnk2* K+ leak channel (TREK-M, [23]) to the hippocampus proper and entorhinal cortex. This study demonstrates the effectiveness of drug-inducible gene therapy to decrease seizures by 90% and highlights the challenges of controlling off-target expression.

## 2. Results

### 2.1. Design of a Drug-Inducible Gene Therapy

**Figure 1:**
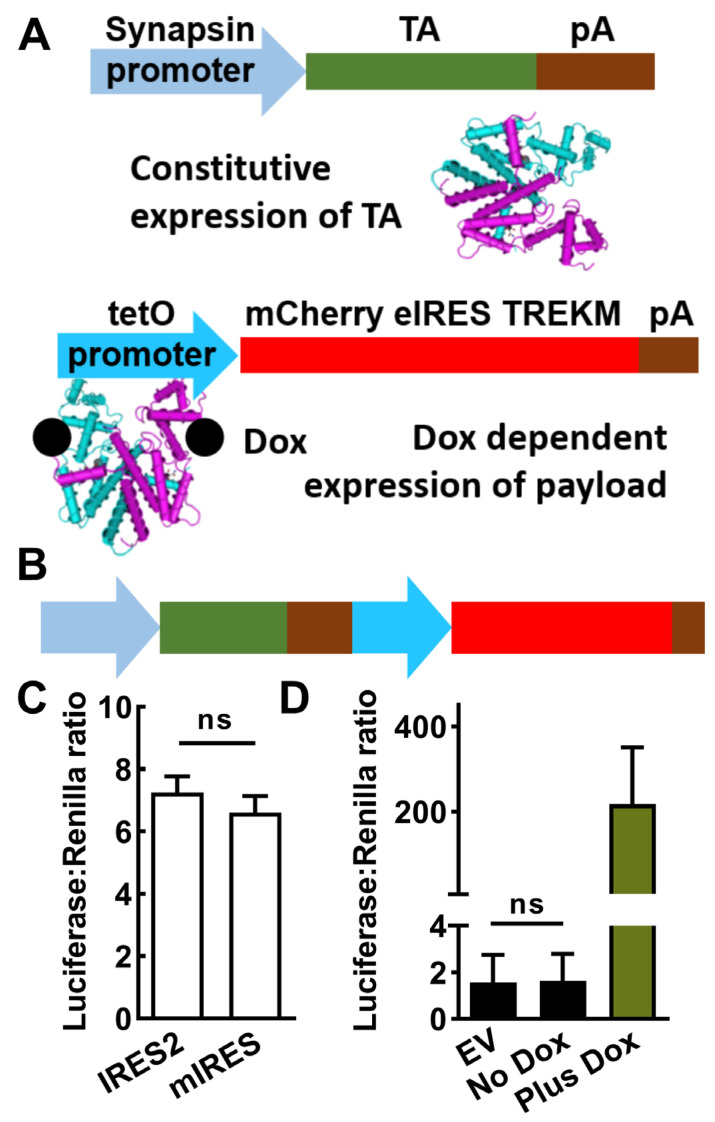
Design and in vitro testing of a drug-inducible gene therapy (SmonCeiT). (**A**), The Dox-On inducible system is composed of two parts: (1) a promoter driving expression of the reverse tetracycline transactivator (TA), and (2) the tet operator promoter (tetO) driving expression of the gene therapy transgene in the presence of Dox. The present study used the neuron-specific human synapsin minimal promoter fragment to drive the TA and the payload included both mCherry and the TREK-1 mutant TREK-M. (**B**), The two elements were combined into a single AAV with the TA element 5′ of the tetO element. (**C**), Comparison of two IRES elements (internal ribosome entry sequences) showing they are equally effective. Activity was measured using a luciferase assay, which uses Renilla luciferase to control for differences in transfection efficiency. Plasmids were based on the pGL backbone (Promega), which used a CMV promoter to drive both mCherry-IRES-luciferase constructs. Results are from three separate transfections, analyzed by Student’s *t*-test (ns, not significantly different). (**D**), In vitro assays of the Synapsin and Dox-On system where the payload was luciferase. No significant difference was observed between empty vector and no Dox using a paired *t*-test (*n* = 4 transfections). The addition of Dox led to a 200-fold increase in luciferase activity.

The Dox-On genetic switch contains two components: (1) the reverse tetracycline transactivator (TA), which in the presence of Dox binds to (2) the tet operator (tetO), which are seven copies of its DNA response element (Figure 1A). Dox-regulated genes are placed downstream of the tetO. These elements were cloned in the following order: synapsin promoter, TA, tetO, and Dox-regulated payload (Figure 1B), which is similar to the first-generation Dox-Off AAV [24]. Three important features of the Dox-On system optimized by Bujard and colleagues [25] are as follows: (1) decreased expression in the absence of Dox (leak); (2) increased expression in its presence and (3) increased potency of Dox to activate expression. A seminal study in rodents showed that a Dox-Off system could be delivered by an AAV and controlled by Dox doses similar to the antibiotic dose used in human patients [24]. The gene we chose to express in a Dox-On gene therapy for epilepsy was a modified K^+^ channel. Previous studies showed that this modified leak K^+^ channel (TREK-M) inhibits neuronal firing and reduces the duration and severity of status epilepticus in rats when delivered via an AAV [23]. For the current proof-of-concept studies, we also expressed the red fluorescent protein, mCherry. One tool to express two genes from a single promoter is to use an Internal Ribosome Entry Sequence (IRES). A widely used IRES is from encephalomyocarditis virus (EMCV). This IRES is ~600 bp long and could not be used due to the size constraints of AAV packaging (~4700 bp). Shorter IRES have been shown to be effective (>300 bp), so we cloned the human IRES from the *eIF4G* gene (eIRES, [26]).

### 2.2. In Vitro Testing of AAV-SmonCeiT

The ability of IRES-2 and eIRES to drive the expression of luciferase in the downstream position (mCherry-IRES-luciferase) was found to be similar (Figure 1C). Luciferase assays were also used to measure synapsin promoter-driven expression of either luciferase (SynLuc) or the Dox-On-driven luciferase (SynDoxLuc). Dox-stimulated expression was 80-fold level higher than the Synapsin-driven luciferase (Figure 1D). However, in the absence of Dox there was no significant luciferase from SynDoxLuc above background empty vector control (EV) while expression in the presence of Dox was robust, boosting expression 1675-fold over background (Figure 1D).

### 2.3. In Vivo Testing of AAV-SmonCeiT

**Figure 2:**
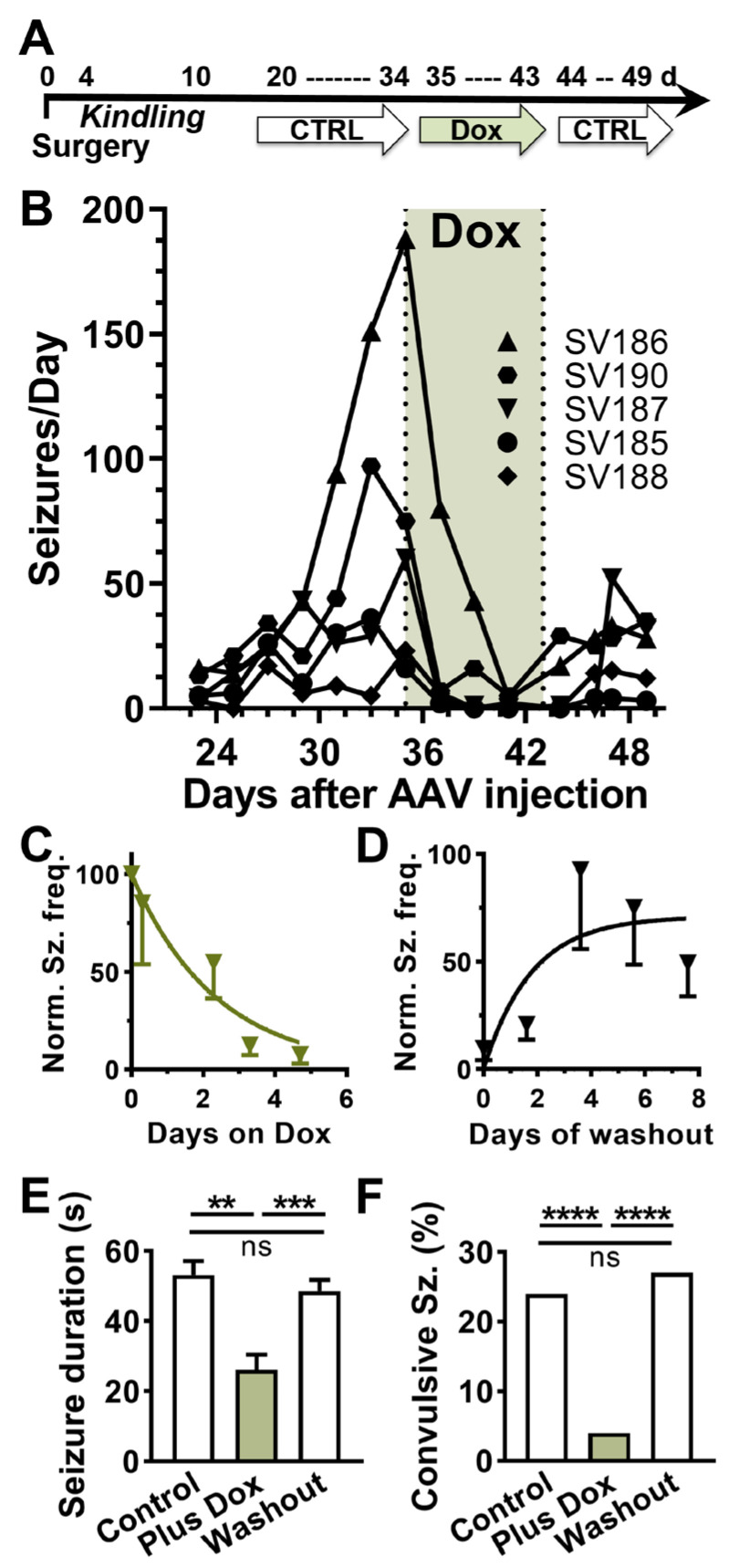
In vivo testing of a Dox-on AAV system in rats. (**A**), Schematic of the experimental time course. AAV-SmonCeiT was injected during the surgery to implant the EEG recording headset. After 4 days of recovery, rats were electrically kindled using a fast kindling protocol (6x/d at 1 h intervals). To allow for AAV expression, we waited 21 days before starting continuous video/EEG monitoring. Spontaneous seizures were observed in all 5 rats. (**B**), Spontaneous seizure frequency (binned in 2-day running average) while rats were fed AIN-93M control chow, Dox containing chow (green shading), washout in control chow. Lines indicate seizure frequencies from individual rats. (**C**,**D**), Kinetics of Dox-On and washout effect on seizure frequencies. The data were normalized to the frequencies observed in the last 3 days on control chow before Dox, and then fit with one-phase exponentials (**C**), K_on_ = 0.4 d^−1^; (**D**), K_off_ = 0.6 d^−1^. (**E**,**F**), Seizure durations (mean ± sem) and the percent of seizures accompanied by tonic-clonic motor component (BSS > 3) from the experiment shown in B (*n* = 5 rats). The treatment periods were defined as follows: Control period, days 31–35; Plus Dox, days 39–43 and Washout, days 45–49. Statistical analysis of duration data used a mixed effects model followed by Tukey’s multiple comparison test. Statistical analysis of the percent convulsive seizures used Fisher’s exact test. Significance shown as: **: *p* < 0.01, ***: *p* < 0.001, ****, *p* < 0.0001.

A useful screen for antiseizure efficacy is testing compounds on evoked seizures in kindled rodents [27]. To apply this assay to test the efficacy of a Dox-On gene therapy, we injected AAV at the time of electrode implantation surgery, electrically-kindled the rats, and then waited 3-weeks post-injection to allow for AAV expression. The original protocol was to test the current required to trigger electrographic seizures (after-discharge threshold; ADT) before and after Dox treatment (Figure 2A). We used convection-enhanced injection to deliver AAV at a dose of 3 × 10^13^ vector genomes per kg brain weight [28]. Unexpectedly, when we restarted the video/EEG recording of the kindled rats, we discovered that they were all spontaneously seizing. Electrographic seizures were accompanied with motor seizures in 24% of cases (BSS > 3532 of 2173 seizures) and seizure frequencies ranging from 20 to 200 seizures per day (*n* = 5; Figure 2B). After two weeks of baseline recording, we switched their chow from a control rodent mature diet AIN-93M to the same AIN-93M diet containing 100 ppm doxycycline. Within days, spontaneous seizures were almost completely abolished (Figure 2B,C; *p* < 0.01, paired *t*-test). After 1-week of Dox treatment, the animals were returned to the control diet, and spontaneous seizures returned (Figure 2D; *p* < 0.05, paired *t*-test). Both the onset and washout of the Dox effect on spontaneous seizures were rapid, occurring over a few days. Dox treatment not only reduced seizure frequency by >90%, but it also significantly reduced the duration and behavioral component of the few seizures that did occur during Dox treatment (Figure 2E,F). Notably, during Dox treatment electrographic seizures were accompanied by motor seizures in only 4% of cases (24 of 658 seizures, *p* < 0.001, Fisher’s exact test). Upon washout, both the seizure duration and fraction of convulsive seizures returned to Control levels (166 of 604 seizures, *p* = 0.14, Figure 2E,F).

**Figure 3:**
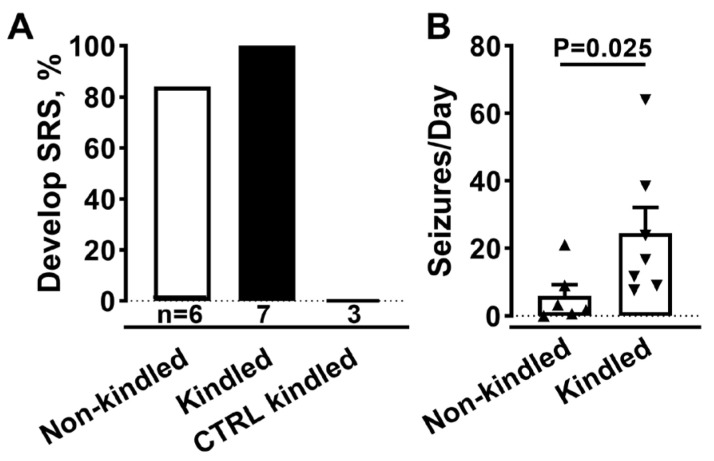
AAV-SmonCeiT triggers SRS in rats in the absence of kindling. (**A**), Rats injected with AAV-SmonCeiT developed seizures whether they were kindled (Kindled, *n* = 7) or not (Non-kindled, 5/6 rats). Rats injected with a control AAV (CTRL) failed to develop seizures after kindling and Dox treatment (*n* = 3). The control AAV was a Dox-on mCherry, it controlled for possible effects of AAV injection, EEG electrodes, expression of the Dox-on components, and expression of mCherry. (**B**), Spontaneous seizure frequencies in AAV-SmonCeiT injected rats that were either not kindled or kindled. Statistical analysis by non-parametric *t*-test followed by Mann-Whitney test.

Spontaneous seizures after AAV injection were observed in three experiments using male Sprague-Dawley rats. As shown in Figure 2, electrical kindling of SmonCeiT-injected rats led to spontaneous seizures with a very high frequency. Therefore, we tested if electrical kindling was required to trigger epilepsy. Indeed, SmonCeiT injection alone was sufficient to cause spontaneous seizures in 5 of 6 rats (Figure 3A).

Rats injected with a control AAV that delivers a Dox-on mCherry did not develop seizures (*n* = 3). A notable difference in SmonCeiT-induced epilepsy was that kindling increased spontaneous seizure frequency 5-fold (Figure 3B, Kindled, 50 ± 10, *n* = 5; Not kindled, 6 ± 3, *n* = 6, *p* < 0.05, unpaired *t*-test).

### 2.4. Evidence for “Leak” Expression in the Absence of Doxycycline

**Figure 4:**
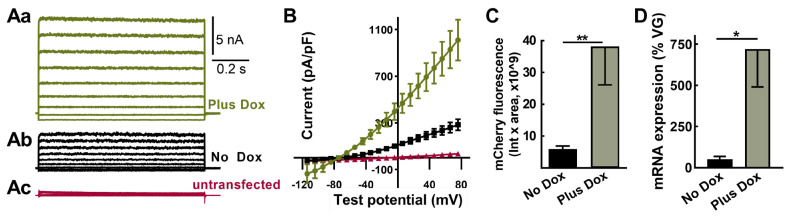
TREK-M and mCherry expression in the absence of Dox can be detected both in vitro and in vivo. (**Aa**–**Ac**), Representative whole cell currents measured after transient transfection of HEK−293 cells in vitro. Transfected cells were visualized using GFP, then recorded either in the presence (**Aa**, green) or absence of Dox (**Ab**, black). Also shown are traces from a cell that did not express GFP (**Ac**, red). (**B**), Average current-voltage relationship from the same three groups (mean ± SEM, *n* = 7, 7, and 4 cells, respectively). (**C**,**D**), Results from rats injected with AAV-SmonCeiT using either fluorescent imaging of brain slices or qPCR. (**C**), mCherry-positive neurons were analyzed in 10x images using cellSens software. Their sum fluorescence was calculated by multiplying the average intensity by the sum area (Plus Dox, 38 ± 12 au × 10^9^, *n* = 8; No Dox, 6 ± 2 au × 10^9^, *n* = 4 rats; *p* < 0.01, Mann-Whitney test). (**D**), Abundance of mCherry and TREK-M mRNAs were estimated by RT-qPCR then normalized to copies of TA detected in PCR of genomic DNA (Plus Dox, 720 ± 230%, *n* = 4 rats; No Dox, 51 ± 16, *n* = 4 rats; *p* < 0.05, Mann–Whitney test. Significance shown as: *: *p* < 0.05, **: *p* < 0.01.

Electrical kindling of rats does not typically result in spontaneous seizures; the exception being “over-kindling” where more stimulations (100–300) were given after reaching the kindled state [29]. A possible explanation for these findings was the leaky expression of TREK-M in the absence of Dox in specific neuronal circuits. Although TREK-M expression was not observed in luciferase assays without Dox (Figure 1D), we re-examined this question with a more sensitive assay: whole-cell patch-clamp electrophysiology. Indeed, a background K^+^ -selective current was readily detectable in HEK-293 cells transfected with SmonCeiT under both No Dox and Plus Dox conditions (Figure 4A). At hyperpolarized potentials, inward K^+^ currents were observed, which reversed at −80 mV and showed only slight outward rectification at positive potentials (Figure 4B). Currents were 5-fold larger in the Plus Dox condition, while untransfected cells showed only small currents with a reversal potential of −24 mV (Figure 4B).

We next examined the Dox regulation of mCherry fluorescence after AAV in vivo injection. Red fluorescent cells with neuronal morphology were readily detected in brain slices from rats treated in either the absence or presence of Dox, and this signal was 6-fold higher in slices from rats treated with Dox (Figure 4C, *p* < 0.01).

We also measured mRNA expression in the hippocampus after in vivo AAV injection using RT-qPCR. To account for variable AAV transduction, we purified genomic DNA and used qPCR to determine AAV vector genomes. Significant mRNA expression was detected in the No Dox condition and was increased 14-fold in animals treated with Plus Dox (Figure 4D, *p* < 0.05).

### 2.5. AAV-SmonCeiT Expression in the Hippocampus

**Figure 5:**
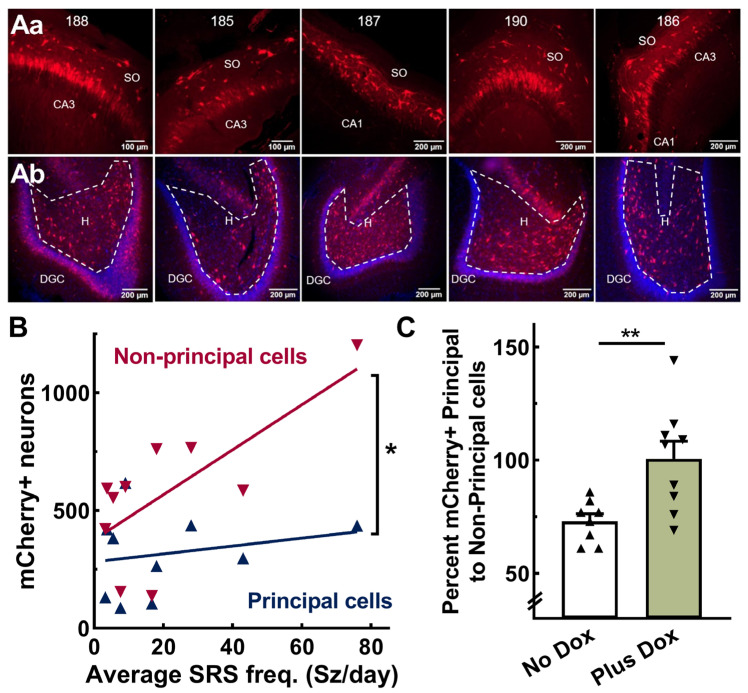
AAV-SmonCeiT expression in the rat hippocampus. (**Aa**,**Ab**), Representative images of horizontal brain slices obtained from the 5 rats shown in Figure 2 (ID #188, 185, 187, 190, 186). Top row (**Aa**) shows images from the CA3 or CA1 pyramidal cell layer including the stratum oriens (SO). Bottom row (**Ab**) shows images of the dentate granule cell layer (DGC; stained blue with DAPI) and hilus (H). mCherry-labeled neurons (mCherry+) were counted in 10× images from every 12th slice. Principal cells were counted in the DGC and CA pyramidal cell layers, while non-principal cells, presumptive inhibitory neurons, were counted in the dentate hilus, stratum oriens, and strata radiatum and lacunosum-moleculare. (**B**), Correlation between spontaneous recurring seizure frequency (SRS) and AAV-SmonCeiT expression in non-principal cells but not principal cells (Pearson correlation analysis, non-pyramidal cells, r = 0.7, *p* = 0.02; pyramidal cells, r = 0.2, *p* = 0.54, *n* = 10 rats). (**C**), Doxycycline treatment increases expression in principal cells to a greater extent than non-principal cells (No Dox, 73 ± 3, *n* = 8; Plus Dox, 101 ± 8, *n* = 9 rats, *p* < 0.01, unpaired *t*-test). Significance shown as: *: *p* < 0.05, **: *p* < 0.01.

Next, we sought to explain the following concurrent phenomena: AAV-SmonCeiT in the No Dox condition caused spontaneous seizures, while in the Plus Dox condition, it abolished seizures. The expression of TREK-M in neurons inhibits neuronal action potential firing by decreasing membrane resistance and hyperpolarizing resting membrane potential [23]. Therefore, the selective expression of TREK-M in inhibitory neurons would tip the excitation:inhibition (E:I) balance towards net excitation. To test this hypothesis, we counted mCherry-positive neurons in subfields of the hippocampus that are enriched in either excitatory (Principal cells) or inhibitory neurons (Non-Principal cells). Figure 5A shows representative images from the cohort used to generate the results in Figure 2. Neuronal counts in hippocampal subfields were pooled as follows: Principal cells included dentate granule cells (DGC) and cornu ammonis (CA) pyramidal cells and Non-principal cells included neurons in the dentate hilus, stratum oriens (SO), stratum lacunosum-moleculare (SLM), and stratum radiatum (SR). Correlation analysis indicated that seizure frequency in the No Dox condition significantly correlated with AAV expression in Non-Principal cells (*p* = 0.02), but not Principal cells (*p* = 0.5; Figure 5B). We then examined if the ratio of Non-Principal to Principal cells differed between No Dox and Plus Dox conditions. Dox treatment significantly increased the detection of mCherry in Principal compared to Non-Principal cells (*p* = 0.01, Figure 5C).

**Figure 6:**
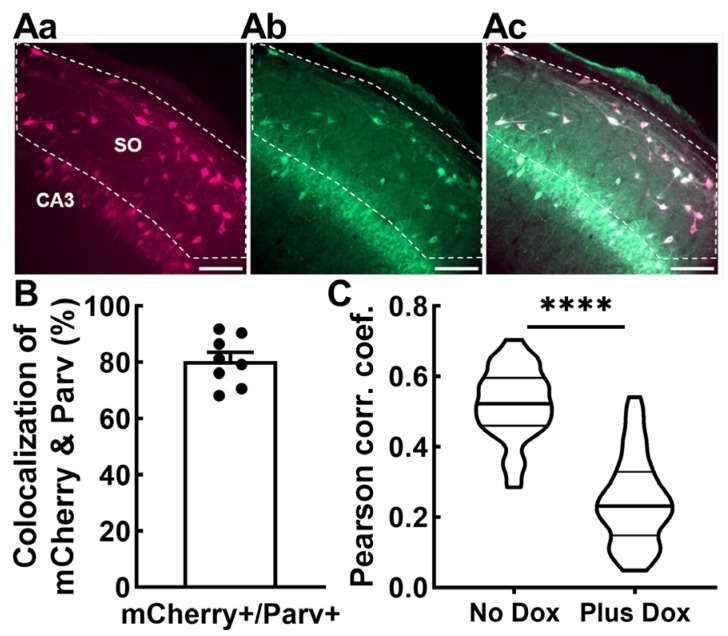
SmonCeiT expression in inhibitory neurons in the rat hippocampus and VGAT-Cre mice. (**Aa**–**Ac**) representative images of anti-mCherry (**Aa**), anti-Parvalbumin (**Ab**), and the overlap (**Ac**). Scale bar represents 100 μm. Confocal imaging was performed at 20× magnification. The “Count and Measure” algorithm in Cellsens was used to identify mCherry+ cells in rat hippocampal dentate hilus and stratum oriens of CA3 and CA1. The detected cells were converted to regions of interest (ROIs), and the anti-parvalbumin signal within the ROI was measured. Cells were considered colocalized if the anti-parvalbumin signal was 1.25× above background. (**B**), Average colocalization measured from epileptic rats shown in Figure 2 and Figure 4. Data obtained from 60 images from eight rats (five kindled No Dox, three non-kindled No Dox). (**C**), Colocalization of AAV-SmonCeiT with a marker of GABAergic inhibitory neurons is higher in the No Dox condition than the Plus Dox condition in VGAT-Cre driver mice. Injection of the Cre-dependent AAV, DIO2xBFP, labeled GABAergic neurons with Blue fluorescent proteins. This signal was colocalized with the Red mCherry fluorescence from SmonCeiT. Colocalization analysis was performed on 51 images taken from two animals who received control chow (8445 cells) and 44 images from two animals who received Dox chow (5882 cells). Violin plots represent Pearson’s correlation coefficients for each image. Statistical analysis included the Kolmogorov–Smirnov test for normality followed by an unpaired *t*-test. Significance shown as: ****: *p* < 0.0001.

Next, we examined whether the Non-Principal cells infected with AAV-SmonCeiT were GABAergic based on colocalization of SmonCeiT with the neuropeptide parvalbumin (Parv), as Parv-expressing hippocampal interneurons are known to be GABAergic and have been implicated as a vulnerable cell population in other rodent epilepsy models [21]. Indeed, neurons expressing mCherry in the dentate hilus and SO of CA3 and CA1 predominantly colocalized with parvalbumin expression in No Dox rats (80.5 ± 3.1%, *n* = 8 rats, 1467 mCherry+ cells, 1163 Parv+ cells; Figure 6A,B). We counted a total of 1532 Parv+ cells, indicating that 75.7 ± 1.7% of them were transfected with SmonCeiT.

To validate these results using an alternative method, we tested AAV-SmonCeiT expression in transgenic mice expressing Cre recombinase in GABAergic neurons (VGAT-Cre; [30]). To label GABAergic neurons, we developed an AAV with Cre-dependent delivery of blue fluorescent proteins and co-injected it with AAV-SmonCeiT. AAV was injected into the dentate hilus, a region with high density of GABAergic neurons that play key roles in suppressing seizures [21]. We compared two groups of injected mice, one that received control No Dox chow and the second that received Plus Dox chow. High-resolution images were then examined for colocalization. Colocalization of AAV-SmonCeiT with AAV-DIO2xBFP was substantially higher in the No Dox group compared to the Plus Dox group, indicating that Dox treatment preferentially increased TREK-M and mCherry expression in excitatory neurons (*p* < 0.001, Figure 6C).

## 3. Discussion

This study highlights both the promise and perils of gene therapy for epilepsy. We show that drug-inducible expression systems can be engineered to fit the size constraints of AAV packaging. To accomplish this size reduction, we developed a short IRES (eIRES) and used minimal SV40 late polyadenylation sequences [31]. For drug-inducibility, we sed the third generation Dox-On gene regulatory system (Clontech Tet-On 3G), which in vitro shows virtually no expression in the absence of Dox and a strong upregulation in its presence [25]. Expression of the Dox transactivator was driven by a short fragment of the human synapsin promoter, which provides strong expression in neurons [32]. We chose drug-inducible delivery of TREK-M, an engineered leak K^+^ channel that was shown to reduce neuronal firing and shorten pilocarpine-induced status epilepticus [23]. We injected the AAV using convection-enhanced delivery [28], allowing AAV to spread down low-resistance channels such as fiber tracts. The dose administered (3 × 10^13^ VG/kg brain weight) can be scaled to human use, making our drug-inducible therapy highly translatable to the clinic. However, while we demonstrate that this putative therapy is highly translatable, off-target effects caused by infecting inhibitory neurons indicate that the neuronal specificity of gene therapy is key when designing gene therapies to combat temporal lobe epilepsy.

A key finding was that electrical kindling of rats injected with AAV-SmonCeiT led to a high frequency of spontaneous recurring seizures. While kindling was not required, it increased seizure frequency. We conclude that kindling acts as a stressor that reveals an underlying propensity for seizure generation. Mathematical models, such as Epileptor [33], predict that a variety of conditions can turn normal behavior into seizures. This concept is also supported by our findings with VGAT-Cre mice, which are phenotypically normal but develop spontaneous seizures after electrical stimulation [34]. Generating rodent models of TLE that are amenable to drug screening has hampered drug development efforts. We suggest that selective inhibition of GABAergic neurons with genetic tools such as SmonCeiT may provide a suitable model to test compounds for antiseizure activity.

A second key finding was that Dox activation of TREK-M expression dramatically decreased the occurrence of spontaneous seizures by over 90%. In addition, the few remaining seizures were more benign in terms of duration and severity based on less pronounced tonic-clonic seizures. Returning the rats to normal chow led to a resumption of the frequency and severity of seizures, indicating that the decline was not due to spontaneous remission. An important characteristic of drug-inducible gene therapies is that they are amenable to cross-over study designs, which increases the statistical power via paired comparisons. Drug development efforts for temporal lobe epilepsy are hampered by animal models that show low frequencies of seizures, clustering, progression in seizure frequency, and spontaneous remission (reviewed in [35]).

Dox activation of expression was robust in both in vitro and in vivo assays, though notably the apparent net fold Dox regulation was much higher in vitro (1000-fold in vitro, 10-fold in vivo). The in vitro assays used the transient transfection of tissue culture cell lines, lasting only 2–3 days. In contrast, in vivo assays were performed weeks after AAV injection, providing time for the slow accumulation of target gene expression in the absence of Dox.

To understand how TREK-M caused seizures in the absence of Dox, we re-examined Dox regulation with patch-clamp electrophysiology. In contrast to luciferase assays, TREK-M currents were readily detectable in the absence of Dox Whole cell currents (I) are directly proportional to the single channel current (i) times the number of channels (N) and the probability of channel opening (Po). Solving this equation with literature values of TREK-1 [36] leads to an estimate that 600 channel molecules are sufficient to cause the 5 nA of current observed in the absence of Dox (Figure 3). No Dox expression was also verified in vivo using imaging assays and qPCR. Future studies are warranted to understand the mechanism of “leaky” TREK-M expression in this Dox-on system to improve the translatability of this preclinical gene therapy.

The synapsin promoter drives neuron-specific expression and shows a slight preference for expression in inhibitory over excitatory neurons [37]. We hypothesized that No Dox expression of TREK-M in inhibitory neurons caused seizures, while Plus Dox expression in all infected neurons abolished seizures. Non-biased cytological analysis revealed higher counts of mCherry-positive neurons located above and below the pyramidal cell layers than within these Principal cell layers. Applying this analysis to all epileptic rats that had been injected with AAV-SmonCeiT revealed a significant correlation between seizure frequency and expression in Non-Principal cells, but no significant correlation to expression in Principal cells. Given the preference of the Synapsin promoter for inhibitory neurons, future studies are warranted to understand if this expression pattern is due to long-range interactions between the synapsin promoter and the tet operator, or due to Dox-independent activity of the reverse tetracycline TransActivator as previously reported by other studies [38]. A limitation to this study is that there are some GABAergic inhibitory neurons located in the pyramidal cell layers, and in the corollary, there are glutamatergic excitatory neurons located outside of pyramidal cell layers, e.g., many Mossy cells in the dentate hilus. Therefore, we tested our hypothesis using two distinct methods: (1) staining epileptic rat slices with an established marker of GABAergic neurons, anti-Parv and (2) colocalizing AAV-SmonCeiT with a Cre-dependent AAV-DIO2xBFP in VGAT-Cre mice. As expected, most of the Non-Principal cells that were labeled by SmonCeiT in rats were Parv-positive in the CA3, CA1, and dentate hilus. In mice fed control chow, most of the mCherry signal from AAV-SmonCeiT was colocalized with BFP-labeled GABAergic neurons. In contrast, in mice fed chow Plus Dox, a greater fraction of mCherry-positive neurons were not labeled with BFP. This result provides strong support for the conclusion of selective inhibition of GABAergic neurons in inhibitory neurons. The conclusion that loss of GABAergic tone can lead to seizures is well supported by studies that tested the GABA hypothesis of epilepsy [21,39].

Although it is possible that the AAV serotype used, rh10, has a higher tropism for GABAergic neurons, a plausible explanation is that both types of neurons were infected and expression remained below the background in excitatory neurons. Preferential expression of the Synapsin promoter in GABAergic neurons provides a plausible explanation for this finding [37]. Finally, this conclusion is supported by gene therapy studies targeting excitatory neurons with a Camk2a promoter, showing reduced seizures in models of epilepsy [14]. A limitation to these studies is that the *Camk2a* gene is also expressed in parvalbumin and somatostatin expressing inhibitory neurons [40], thereby causing off-target effects as seen in this study.

## 4. Materials and Methods

### 4.1. Molecular Cloning

A TREK-1 mutant channel (TREK-M) was cloned as previously described [23]. TREK-M contains mutations to reduce second messenger regulation and stabilize the open state of the channel (S333A, S300A, E307A). For expression in AAV, we replaced the lentiviral long-terminal repeats in pLVUTHM (a gift from Patrick Aebischer and Didier Trono, Addgene plasmid 11650), with synthetic AAV2 internal terminal repeats [41,42]. For doxycycline regulation, we cloned in the reverse tetracycline transactivator (TA) and its binding site (tet operator, tetO) from Tet-on 3G plasmids (Takara, Mountain View, CA, USA). Cloning steps used oligonucleotide primers to the gene target that added unique restriction enzyme sites. The Dox-regulated payload includes both TREK-M and mCherry (a gift from the late Roger Y. Tsien). To express both proteins from a single mRNA, we cloned a short Internal Ribosome Entry Sequence (IRES) derived from the human eIF4G gene [26]. The 264-bp eIRES corresponds to nucleotides 107–370 of GenBank accession number NM_004953. In vitro, testing showed eIRES worked as well as the commonly used 587-bp IRES from encephalomyocarditis virus. To drive AAV expression in neurons, we cloned in the human 0.5-kb human synapsin promoter [32]. Synapsin promoter activity was enhanced by adding the 124-bp minimal CMV promoter [25] as described previously for the GABRA4 promoter [23]. The resulting AAV targeting plasmid was abbreviated SmonCeiT to signify Synapsin CMV minimal promoter driving Dox-on expression of mCherry-eIRES-TREK-M. This plasmid was used for packaging into an adeno-associated virus (AAV serotype rh10; AAV10) and titered by the University of Pennsylvania Vector Core (5.5 × 10^12^ Vector Genomes/mL (VG). The negative control virus used a similar design (abbreviated EonG3dC), except it used a 0.24-kb rat enolase promoter to drive the Dox-on components and did not contain either eIRES or TREK-M.

We replaced the double-inverted open reading frame in pAAV-EF1a-double floxed-hChR2-EYFP-WPRE-HGHpA (gift from Karl Deisseroth; Addgene plasmid #20298) with two BFPs separated by a P2A linker. The subsequent construct then contained the two BFPs in inverse orientation from the EF1a promoter. Cre recombinase expression in VGAT-Cre mice [30] reverses the orientation of the BFP cassette and allows expression in GABAergic neurons. Tag-BFP was a gift from Dawen Cai and Joshua Sanes (Addgene plasmid # 45185; [43]) and EBFP2-Nuc was a gift from Robert Campbell (Addgene plasmid # 14893; [44]). AAV9-DIO2xBFP viral particles were prepared by Vigene (1 × 10^13^ vector genomes/mL; VG).

### 4.2. Stereotactic Injection of AAV and Implantation of Electrodes

The animal protocols were approved by the Animal Care and Use Committee at the University of Virginia and follow ARRIVE guidelines [45]. Fifty-day-old male Sprague Dawley rats were obtained from Charles River Laboratories (Wilmington, MA, USA), and were acclimated to the temperature and humidity-controlled vivarium for at least 48 h before beginning surgeries. Only male rats were used to exclude the effects of female hormone cycling on seizure frequency [46]. Rats were given ad libitum access to food and water. Rats were anesthetized using 2–3% isoflurane and given eye lubricant and placed on a heating pad to ensure the animal maintained proper body temperature. Animals were then placed into a stereotactic frame (Model 940 with digital display, David Kopf Instruments, Tujunga, CA, USA). AAV was injected using the following stereotactic coordinates (mm from bregma): for CA3, Lateral 5.1, Anterior-Posterior 5.4 (CA3) and for the subiculum and entorhinal cortex junction, Lateral 4.6, Anterior-Posterior 7.8 [47]. Using a Hamilton syringe, 3 µL of AAV10-SmonCeiT at a rate of 20 nl/s was injected at each of these locations using a UMP3 microsyringe Injector and Micro4 controller (World Precision Instruments, Sarasota, FL, USA). Injections began at the deepest injection location and gradually raising the needle to the shallowest depth of each injection site (from bregma, 7.6 to 4.6 mm). The needle remained in place at each injection site for 2 min following each injection to prevent backflow of AAV. The stimulating depth electrodes were made of 0.008” diameter perfluoroalkoxy-coated stainless steel wire (A-M Systems, Sequim, WA, USA). They were placed in the CA3 coordinates into the left hemisphere at a depth of 6 mm below bregma (Channel 1 and 2). We also implanted two bilateral supradural cortical electrodes (Channels 3 and 4) and a supradural cerebellar reference electrode (Channel 5). The EEG headset was secured in place using dental acrylic cement. Rats were given ketoprofen and bupivacaine to provide analgesia following surgery. Rats were allowed to recover from surgery for one week prior to electrical kindling.

### 4.3. Kindling and Electroencephalogram (EEG) Recordings

Kindling used electrical stimulation protocols described previously [48,49]. This procedure involved six daily 10-s pulse trains at 50 Hz each with an amplitude of 400 μA. The current was delivered via the depth electrode using a constant current amplifier (Model 2100, A-M Systems, Sequim, WA, USA). Behavioral seizure scoring (BSS) used a modified Racine scale as follows [50]: BSS 0, no detectable change in behavior; BSS 1, behavioral arrest; BSS 2, facial twitching, head bobbing; BSS 3, unilateral forelimb clonus; BSS4, bilateral forelimb clonus; BSS 5, rearing and falling and BSS 6, running and involuntary jumping. Kindling was performed until rats were considered “fully kindled,” which was defined as five consecutive tonic-clonic seizures in response to stimulation. After kindling, rats were returned to their home cage for 10 d. Rats were then connected to a video-EEG monitoring system composed of Grass amplifiers and Stellate Systems software (Montreal, Canada) via a low torque commutator. Video/EEG was monitored 24 h/d, 7 d/wk. Animals were fed American Institute of Nutrition Rodent Diet 93M (AIN-93M; [51]) chow in white pellets during control periods and beige pellets containing 100 ppm doxycycline to activate the Dox-On gene therapy (Research Diets, Inc, New Brunswick, NJ, USA), as a similar Dox concentration was previously shown to modulate Dox-regulated gene delivery in the brain [24].

### 4.4. Imaging Studies of AAV Distribution

Rats were injected with pentobarbital and then transcardially perfused using freshly prepared 4% paraformaldehyde solution (PFA in PBS). Following perfusion, brains were removed and stored at 4 °C in 4% PFA for 24 h, followed by embedding brains in low melting point agarose and slicing them into 40 μm-thick horizontal sections using a Leica VT 1000S vibratome (Leica Biosystems, Buffalo Grove, IL, USA). Sections were stored in cryoprotectant (30% glycerol, 30% ethylene glycol, 0.2 M phosphate buffer) at −20 °C until mounting.

Imaging of native mCherry fluorescence was performed on every 12th horizontal brain slice from each animal, corresponding to −4 to −8.5 mm below bregma. Slices were mounted on slides with Fluoromount-G plus DAPI (Southern Biotech, Birmingham, AL, USA). A reference atlas was used to define the following subfields for subsequent imaging and analysis: dentate granule cell layer, dentate hilus, CA pyramidal cell layers, stratum oriens, stratum radiatum, subiculum, and entorhinal cortex [47]. Images were acquired with a 10× objective using an Olympus IX 81 microscope equipped with appropriate fluorescent cubes (Olympus, Tokyo, Japan). Imaging and analysis used Olympus cellSens software (RRID:SCR_014551). Regions of interest (ROI) were drawn around each subfield to be analyzed, and neurons were counted using the Count and Measure module within cellSens. All analyses were performed by investigators blinded to the experimental condition of each rat.

### 4.5. DNA and RNA Isolation and Real Time Quantitative PCR (RT-qPCR) Protocols in Rats

RT-qPCR studies were performed on eight rats that were injected with AAV but were not implanted with EEG headsets. They were fed for 4 weeks either control AIN-93M chow (*n* = 4) or AIN-93M plus 100 ppm doxycycline (*n* = 4). In order to isolate DNA and RNA, rats were euthanized via decapitation. Gross dissection of the hippocampal formation and entorhinal cortex was then performed on a 4 °C stage covered in saline, placed into Trizol reagent, and then lysed using a Polytron homogenizer (Kinematica AG, Malters, Switzerland). Following phase separation and prior to binding, half of the sample was used for DNA isolation (Isolate II Genomic DNA kit, Bioline, London, UK) and the other half was used for total RNA isolation (Purelink RNA mini kit, Ambion, Austin, TX, USA). cDNA was formed from the isolated RNA using the iScript cDNA Synthesis Kit (Bio-Rad, Hercules, CA, USA). RT-PCR was performed using a SensiFAST SYBR and Fluorescein Kit (Bioline) and a Biorad MyiQ thermocycler (RRID:SCR_019736). The sequences of all primers are shown in Table 1. Primers were designed using NCBI’s Primer-BLAST [52]. Primer efficiencies were verified to be >90%. PCR products were sequenced to verify correct targeting (Genewiz, South Plainfield, NJ, USA).

### 4.6. Histology of Parvalbumin-Expressing Hippocampal Interneurons in Rats

Immunohistochemistry was performed on hippocampal sections from a subset of eight epileptic rats used to visualize AAV-SmonCeiT expression. Anti-parvalbumin (Parv) mouse monoclonal (clone PARV-19, Sigma-Aldrich, St. Louis, MO, USA, Cat# SAB4200545, RRID:AB_2857970, 1:1000) was used to label GABAergic neurons with a secondary Alexa Fluor 647-conjugated goat anti-mouse IgG1 (SouthernBiotech Birmingham, AL, USA, Cat# 1071-31, RRID:AB_2794429). To ensure detection of mCherry, its signal was amplified by staining with anti-mCherry (Thermo Fisher Scientific Pittsburgh, PA, USA, Cat# PA5-34974, RRID:AB_2552323) and an Alexa Fluor 568-conjugated goat anti-rabbit (Thermo Fisher Scientific Cat# A78955, RRID:AB_2925778). Confocal imaging methods were as described above, except images were collected with a 20× objective.

### 4.7. Cre-Dependent AAV-SmonCeiT Expression in Mice

VGAT-Cre mice, which have an IRES-Cre recombinase cassette knocked into the vesicular GABA transporter gene (RRID:IMSR_JAX:028862; [30]) were used to examine the expression of AAV-SmonCeiT in GABAergic neurons. These studies were performed on VGAT-Cre mice that were only injected with AAVs. They were fed either control AIN-93M chow or AIN-93M plus 100 ppm doxycycline (*n* = 4). To visualize GABAergic neurons, we coinjected AAV-SmonCeiT with the Cre-dependent AAV-DIO2xBFP into the dentate hilus, then waited 4 weeks for expression. Confocal imaging was performed using a 20× objective on both red and blue fluorescent protein channels. Images were analyzed using the colocalization module in cellSens.

### 4.8. Statistical Analysis

Results were imported into Excel, averaged, and then imported into GraphPad Prism for statistical analysis ((San Francisco, CA, USA, RRID:SCR_002798). The data were first tested for normality using the Shapiro–Wilk or Kolmogorov–Smirnov tests with α = 0.05. If the data passed the normality tests, the following parametric tests were used: Student’s two-tailed *t*-test and ordinary one-way ANOVA followed by Tukey’s multiple comparisons test. The non-parametric Mann–Whitney U test and Welch’s ANOVA were used in cases of non-normally distributed data. Brown–Forsythe and Bartlett’s tests were used to determine if the standard deviation (SD) of distributions were significantly different. If distributions were significantly different, then a Dunnett’s T3 multiple comparison test was used; otherwise, an uncorrected Fisher’s test was used. One-phase exponential fits were used to determine the kinetics of the Dox-On and Dox-Off effects on seizure frequency. Statistical analysis of seizure duration across a treatment period and across animals used a mixed effects model followed by Tukey’s multiple comparison test. Since behavioral scores are nominal data, we used a nonparametric ANOVA followed by the Friedman test. Pearson’s r was used to calculate statistically significant correlations between spontaneous recurring seizure frequency and numbers of mCherry-positive, hippocampal neurons. For all analyses, statistical significance was determined using α = 0.05. Statistical tests used are noted in the figure legends.

## 5. Conclusions

These studies demonstrate that drug-inducible gene therapies are effective in reducing spontaneous seizures in rats and highlight the importance of testing for side effects with pro-epileptic stressors such as electrical kindling. These studies also show the importance of evaluating the location and spread of AAV-based gene therapies in preclinical trials for novel antiseizure medications in order to prevent any off-target side effects.

## Figures and Tables

**Table 1:** qPCR Primer sequences.

Target	Orientation	Sequence (5′-3′)
TREK-1	F	TCCTCTTTGTGGCTCTCCCT
TREK-1	R	ACACCTCGTTCTCGTAGCAG
mCherry	F	CTCCGACGGCCCCGTAATGC
mCherry	R	CGATGGTGTAGTCCTCGTTG
TA	F	CCGCCGTGGGCCACTTTACA
TA	R	ATCGTCAAGGGCGTCGGTCG

## Data Availability

The data presented in this study are available in this article and upon request.
